# KEDformer: Knowledge extraction seasonal trend decomposition for long-term sequence prediction

**DOI:** 10.1371/journal.pone.0335047

**Published:** 2025-10-24

**Authors:** Zhenkai Qin, Baozhong Wei, Caifeng Gao, Jianyuan Ni

**Affiliations:** 1 School of Information Technology, Guangxi Police College, Guangxi, China; 2 Department of Information Technology and Computer Science, Juniata College, Huntingdon, Pennsylvania, United States of America; National University of Sciences and Technology NUST, PAKISTAN

## Abstract

Time series forecasting is essential in energy, finance, and meteorology. However, existing Transformer-based models face challenges with computational inefficiency and poor generalization for long-term sequences. To address these issues, this study proposes the KEDformer framework. It integrates knowledge extraction and seasonal-trend decomposition to optimize model performance. By leveraging sparse attention and autocorrelation, KEDformer reduces computational complexity from O(L^2^) to O(L log L), enhancing the model’s ability to capture both short-term fluctuations and long-term patterns. Experiments on five public datasets covering energy, transportation, and weather tasks demonstrate that KEDformer consistently outperforms traditional models, with an average improvement of 10.4% in MSE prediction accuracy and 2.9% in MAE prediction accuracy.

## 1 Introduction

Long-term forecasting plays a critical role in decision-making domains such as transportation logistics [1], healthcare monitoring [2,3], utility management [4,5], and energy optimization [6,7]; however, as the forecasting horizon increases, so do computational demands and the complexity of modeling temporal dependencies. Traditional time series decomposition methods, while useful, often rely on linear assumptions, limiting their effectiveness in handling complex multivariate scenarios or unpredictable, non-stationary data patterns, and struggling to capture the interplay between components such as trends, seasonality, and irregularities [8]. Recent advancements have integrated deep learning approaches into the decomposition process to improve forecasting accuracy [9,10], leveraging representation learning and nonlinear transformations to better capture dynamic dependencies and multi-scale interactions within time series data [11,12]. More recently, transformers have excelled in various tasks, such as computer vision (CV) [13,14], natural language processing (NLP) [15], and time series forecasting, due to their powerful modeling capabilities and flexibility, but in long-term forecasting tasks, they face significant challenges. The computational complexity of the traditional self-attention mechanism is *O*(*L*^2^), where *L* represents the sequence length, leading to an increasing demand for memory and computational resources and limiting their applicability in resource-constrained or real-time analysis scenarios. To address this, many researchers have improved the self-attention mechanism to reduce the computational complexity of long-term forecasting and enhance the application of models in practical scenarios [16–19]; additionally, transformers often struggle to model long-term dependencies effectively due to noise interference, where irrelevant information weakens the attention distribution and degrades overall performance [19,20]. Consequently, capturing long-term dependencies in time series data while ensuring computational efficiency over extended prediction horizons remains a significant challenge.

To address the challenges in long-term time series forecasting, we propose an end-to-end Knowledge Extraction Decomposition (KEDformer) framework. The core of this framework is the Knowledge Extraction Attention module (KEDA, blue block), which reduces model parameters through autocorrelation and sparse attention mechanisms. The autocorrelation mechanism estimates the correlations of subsequences within a specific time period, while the sparse attention filters the weight matrix of these correlations, thereby reducing computational overhead and mitigating the interference from irrelevant features. This design reduces the computational complexity from O(L^2^) to O(LlogL), significantly decreasing memory usage and enhancing the model’s ability to process long sequences. Additionally, KEDformer integrates a mixed time series pooling decomposition method (MSTP, yellow block) that decomposes the input time series data into seasonal and trend components, further improving prediction accuracy. This approach captures both short-term fluctuations and long-term patterns, making the predictions more consistent with real-world temporal dynamics. Therefore, the KEDformer framework not only addresses the computational bottleneck of traditional Transformers in long-term forecasting but also enhances their performance and robustness in complex sequence tasks. In summary, the contributions of this study are as follows:

- We introduce a knowledge extraction mechanism that combines sparse attention and autocorrelation to reduce the computational cost of the self-attention layer. This mechanism reduces the computational overhead from quadratic to linear complexity.
- Furthermore, by employing seasonal-trend decomposition, KEDformer effectively captures both long-term trends and seasonal patterns, overcoming the limitations of the Transformer model in capturing long-term dependencies.
- Extensive experiments on five public datasets demonstrate the effectiveness and competitiveness of the proposed KEDformer, which outperforms all previous Transformer-based models across various forecasting applications.

## 2 Related work

### 2.1 Transformer-based long-term time series forecasting

Transformer-based models have demonstrated exceptional performance in time series forecasting due to their powerful self-attention mechanism and parallel processing capabilities, excelling at capturing long-term dependencies and handling long-sequence data [21,22]; however, traditional Transformer models still face several challenges in time series forecasting, such as high computational complexity and difficulty addressing noise issues in long-term dependencies [15,18], for instance, the core self-attention mechanism exhibits quadratic computational complexity with respect to sequence length, which limits its efficiency in long-sequence tasks [23].

To overcome these limitations, various advancements have been proposed in recent years. For example, Synthesizer [24] investigated the importance of dot-product interactions, introduced randomly initialized, learnable attention mechanisms, and demonstrated competitive performance in specific tasks. Furthermore, FNet [25] replaced self-attention with Fourier transforms, showcasing its effectiveness in mixing sequence features. Another approach utilized Gaussian distributions to construct attention weights, enabling a focus on local windows and improving the performance of models in capturing local dependencies [26]. Pyraformer employed a pyramid attention structure to address the complexity of handling long-range dependencies, while TFT integrated multivariate features and time-varying information to improve multi-step forecasting [27]. More recently, Informer introduced the ProbSparse attention mechanism and distillation techniques, reducing computational complexity to O(LlogL) and significantly improving efficiency. LogTrans employed logarithmic sparse attention to further alleviate the computational burden of long-sequence predictions [28], while AST combined adversarial training and sparse attention to enhance robustness in complex scenarios [29];ACSAformer [30] enhances the modeling capability for complex high-dimensional data by integrating sparse attention and adaptive graph convolution.SFDformer [31] improves the modeling and prediction of complex dynamic features by integrating Fourier transform, time series decomposition, and sparse attention mechanisms.Additionally, Autoformer leveraged time series decomposition and autocorrelation mechanisms for long-term sequence forecasting [16,32], and FEDformer utilized frequency-domain enhancements to optimize performance on long sequences [29,33]. We summarize the main characteristics and advantages of some published studies in the literature in Table 1. Although these studies have made progress in optimizing computational efficiency and capturing long-term dependencies, they still show instability in modeling complex long-term and non-periodic dependencies.

**Table 1:** Comparison of transformer-based time series forecasting models.

Model	Main Features	Advantages
Transformer	Based on the self-attention mechanism to capture correlations in sequence data.	Simple model structure, easy to implement, and capable of capturing long-term dependencies.
LogSparse Transformer	Uses causal convolutions to implement convolutional self-attention.	Improves prediction accuracy for fine-grained, long-term dependencies under limited memory.
Informer	Employs ProbSparse self-attention mechanism and self-attention distillation.	Significantly reduces computational complexity and avoids cumulative errors.
Autoformer	Based on time series decomposition and autocorrelation mechanisms.	Reduces computational complexity and enhances performance for long-term sequence forecasting.
Pyraformer	Uses a pyramid attention structure.	Addresses the complexity of handling long-term dependencies.
LogTrans	Uses logarithmic sparse attention.	Further alleviates the computational burden for long-sequence predictions.
FEDformer	Utilizes frequency-domain enhancements.	Optimizes performance on long sequences.

Unlike previous studies, the proposed KEDformer integrates sparse attention mechanisms with autocorrelation strategies at the architectural level and introduces a dominant weight distribution selection mechanism. This mechanism dynamically selects representative weights from the sparse attention matrix, thereby enhancing the model’s ability to capture key dependencies in time series. It not only inherits the computational efficiency of sparse attention but also alleviates the potential information loss often seen in traditional sparse schemes when modeling global contexts. Compared with Informer, which implements sparse attention through probabilistic sampling, KEDformer combines dominant distribution selection with explicit autocorrelation enhancement to improve the stability of modeling long-range, non-periodic dependencies. Unlike Autoformer, which entirely replaces attention with autocorrelation, KEDformer retains the sparse attention backbone and embeds auxiliary autocorrelation modules, forming a complementary modeling framework. This collaborative fusion strategy achieves a balance between performance and efficiency, demonstrating structural innovation and practical adaptability beyond existing methods.

### 2.2 Decomposition of time series

Time series decomposition is a traditional approach that breaks down time series data into components such as trend, seasonality, and residuals, revealing the intrinsic patterns within the data [34,35]. Among traditional methods, ARIMA [36] uses differencing and parameterized modeling to decompose and forecast non-stationary time series effectively. In contrast, the Prophet model combines trend and seasonal components while accommodating external covariates [29], making it suitable for modeling complex time series patterns. Matrix decomposition-based methods, such as DeepGLO, extract global and local features through low-rank matrix decomposition, while N-BEATS employs a hierarchical structure to dissect trends and periodicity [35]. However, these approaches primarily focus on the static decomposition of historical sequences and often fall short in capturing dynamic interactions for future forecasting.

More recently, deep learning models have increasingly incorporated time series decomposition to enhance predictive power. For example, Autoformer introduces an embedded decomposition module, treating trend and seasonal components as core building blocks to achieve progressive decomposition and forecasting [37]. FEDformer combines Fourier and wavelet transforms to decompose time series into components of varying frequencies, capturing global characteristics and local structures while significantly reducing computational complexity and improving the accuracy of long-sequence predictions. Similarly, ETSformer [38] adopts a hierarchical decomposition framework inspired by exponential smoothing, segmenting time series into level, growth, and seasonality components [39]. By integrating exponential smoothing attention and frequency-domain attention mechanisms, ETSformer effectively extracts key features, demonstrating superior performance across multiple datasets [40]. Inspired by these studies, our proposed KEDformer approach integrates decomposition modules dynamically with a progressive decomposition strategy, not only significantly improving computational efficiency but also enabling the simultaneous modeling of both short-term and long-term patterns.

## 3 Methodology

### 3.1 Background

The Long Sequence Time Forecasting (LSTF) problem is defined within a rolling forecasting setup, where predictions over an extended future horizon are made based on past observations within a fixed-size window [23]. At each time point *t*, the input sequence ${𝒳}^{t}=\{x_{1}^{t},\ldots ,x_{L_{x}}^{t}\}$ consists of observations with multiple feature dimensions, and the output sequence ${𝒴}^{t}=\{y_{1}^{t},\ldots ,y_{L_{y}}^{t}\}$ predicts multiple future values. The output length *L*_*y*_ is intentionally set to be relatively long to capture complex dependencies over time. This setup enables the model to predict multiple attributes, making it well-suited for time series applications.

### 3.2 Data decomposition

In this section, we will cover the following aspects of KEDformer: (1) the decomposition process designed to capture seasonal and trend components in time series data; and (2) the architecture of the KEDformer encoder and decoder.

**Fig 1:**
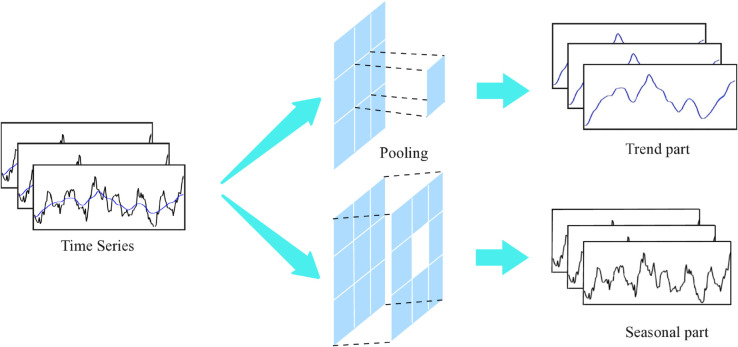
Schematic diagram of mixed time series pooling decomposition block.

**Time series decomposition.** To capture complex temporal patterns in long-term predictions, we utilize a decomposition approach that separates sequences into trend, cyclical, and seasonal components. These components correspond to the long-term progression and seasonal variations inherent in the data. However, directly decomposing future sequences is impractical due to the uncertainty of future data. To address this challenge, we introduce a novel internal operation within the sequence decomposition block, referred to as the autocoupling mechanism in KEDformer, as shown in Fig 1. This mechanism enables the progressive extraction of long-term stationary trends from predicted intermediate hidden states. Specifically, we adjust the moving average to smooth periodic fluctuations and emphasize the long-term trends. For the length-*L* input sequence $\tilde{\chi }\in {\mathbb{R}}^{L\times d}$, the procedure is as follows:

$$x_{t}=AvgPool(Padding(X))$$ (1)

$$x_{s}=x-x_{t}$$ (2)

where $x_{s},x_{t}\in {\mathbb{R}}^{L\times d}$ represent the seasonal part and the extracted trend component, respectively. We use AvgPool(·) for moving average and filling operations to maintain a constant sequence length. We summarize the above process as $x_{s},x_{t}=\text{MSTPDecomp}(x)$, which is a within-model block.

#### Model input.

**Fig 2:**
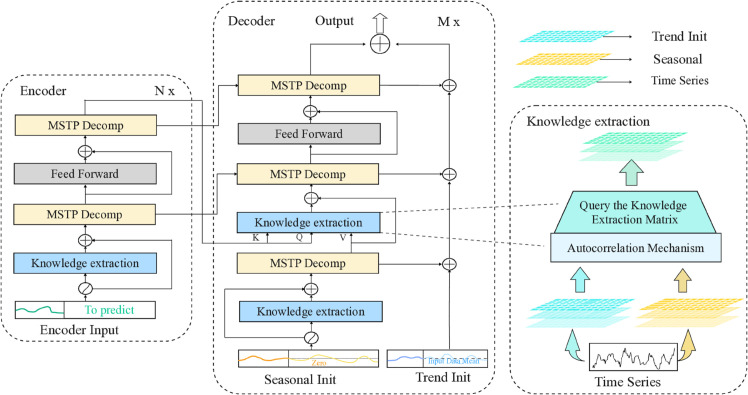
Schematic overview of the proposed. KEDformer method. Knowledge Extraction Attention module (KEDA, blue block), Mixed time series pooling decomposition (MSTP, yellow block).

In Fig 2, the encoder’s input consists of the past *I* time steps, denoted as $X_{\text{en}}\in {\mathbb{R}}^{L\times d}$. In the decomposition architecture, the input to the decoder is composed of both a seasonal component, $X_{\text{des}}\in {\mathbb{R}}^{(I/2+O)\times d}$, and a trend-cyclical component, $X_{\text{det}}\in {\mathbb{R}}^{(I/2+O)\times d}$, both of which are subject to further refinement. Each initialization consists of two elements: (1) the decomposed component derived from the latter half of the encoder’s input, $X_{\text{en}}$, of length *I*/2, which provides recent information, and (2) placeholders of length *O*, filled with scalar values. The formulation is as follows:

$${𝒳}_{\text{ens}},{𝒳}_{\text{ent}}=\mathrm{MSTPDecomp}\left({𝒳}_{\text{en}\frac{I}{2}:I}\right)$$ (3)

$${𝒳}_{\text{des}}=\mathrm{Concat}\left({𝒳}_{\text{ens}},{𝒳}_{0}\right)$$ (4)

$${𝒳}_{\text{det}}=\mathrm{Concat}\left({𝒳}_{\text{ent}},{𝒳}_{\text{Mean}}\right)$$ (5)

where $X_{\text{ens}},X_{\text{ent}}\in {\mathbb{R}}^{\frac{I}{2}\times d}$ denote the seasonal and trend-cyclical components of $X_{\text{en}}$, respectively. The placeholders, labeled as $X_{0},X_{\text{Mean}}\in {\mathbb{R}}^{O\times d}$, are populated with zeros and the mean values of $X_{\text{en}}$, respectively.

#### Encoder.

In Fig 2, the encoder follows a multilayer architecture, defined as $X_{\text{en}}^{l}=\text{Encoder}(X_{\text{en}}^{l-1}),$ where l∈{1,…,N} represents the output of the *l*-th encoder layer. The initial input, $X_{\text{en}}^{0}\in {\mathbb{R}}^{L\times d}$, corresponds to the embedded historical time series. The Encoder function, Encoder(·), is formally expressed as:

$${𝒮}_{\text{en}}^{l,1}=\text{ MSTPDecomp}\left(\mathrm{KEDA}\left({𝒳}_{\text{en}}^{l-1}\right)+{𝒳}_{\text{en}}^{l-1}\right)$$ (6)

$${𝒮}_{\text{en}}^{l,2}=\text{ MSWTDecomp}\left(\mathrm{FeedForward}\left({𝒮}_{\text{en}}^{l,1}\right)+{𝒮}_{\text{en}}^{l,1}\right)$$ (7)

$${𝒳}_{\text{en}}^{l}={𝒮}_{\text{en}}^{l,2}$$ (8)

where $S_{\text{en}}^{l,i}$, i∈{1,2} represents the seasonal component after the *i*-th decomposition block in the *l*-th layer.

#### Decoder.

In Fig 2, the decoder has two roles: the accumulation of the trend time series part and the knowledge extraction stacking of the seasonal time series part. For example, $x_{\text{de}}^{l},\tau_{\text{de}}^{l}=\text{Decoder}\left(x_{\text{de}}^{l-1},\tau_{\text{de}}^{l-1}\right),$ where l∈{1,…,N} represents the output of the *l*-th decoder layer. The decoder is formalized as:

$${𝒮}_{de^{\prime }}^{l,1}\tau_{de}^{l,1}=\text{ MSWTDecomp}\left(\mathrm{KEDA}\left({𝒳}_{de}^{l-1}\right)+{𝒳}_{de}^{l-1}\right)$$ (9)

$${𝒮}_{de^{\prime }}^{l,2}\tau_{de}^{l,2}=\text{ MSWTDecomp}\left(\mathrm{KEDA}\left({𝒮}_{de^{\prime }}^{l,1},{𝒳}_{en}^{N}\right)+{𝒮}_{de}^{l,1}\right)$$ (10)

$${𝒮}_{de^{\prime }}^{l,3}\tau_{de}^{l,3}=\text{ MSWTDecomp}\left(\mathrm{FeedForward}\left({𝒮}_{de}^{l,2}\right)+{𝒮}_{de}^{l,2}\right)$$ (11)

Eq (11) denotes the final decomposition stage within the decoder, where the output from the previous residual block, $S_{de}^{l,2}$, is passed through a feed-forward layer and added back via a residual connection. The resulting sequence is then processed by the MSWTDecomp function, which applies multi-scale wavelet transform-based decomposition to extract seasonal ($S_{de}^{l,3}$) and trend ($T_{de}^{l,3}$) components. This design ensures that each decoder layer can refine the representation by isolating frequency-aware patterns, thereby improving long-horizon forecasting accuracy.

$$\chi_{de}^{l}={𝒮}_{de^{\prime }}^{l,3}$$ (12)

Eq (12), the final seasonal component $S_{de^{\prime }}^{l,3}$—obtained from the MSWTDecomp operation—is directly used as the decoder’s output representation $\chi_{de}^{l}$. This substitution simplifies the decoder architecture by avoiding explicit trend aggregation and emphasizes the dominant short-term patterns captured via multi-scale decomposition, which are particularly useful for downstream forecasting tasks.

$$\tau_{de}^{l}=T_{de}^{l-1}+{𝒲}_{l,1}\cdot {𝒯}_{de}^{l,1}+{𝒲}_{l,2}\cdot {𝒯}_{de}^{l,2}+{𝒲}_{l,3}\cdot {𝒯}_{de}^{l,3}$$ (13)

In this context, $S_{\text{de}}^{l,i}$ and $T_{\text{de}}^{l,i}$, where i∈{1,2,3}, represent the seasonal and trend components, respectively, after the *i*-th decomposition block within the *l*-th layer. The matrix *W*_*i*,*L*_, where i∈{1,2,3}, serves as the projection matrix for the *i*-th extracted trend component, $T_{\text{de}}^{l,i}$.

To improve the flexibility of trend modeling, the decoder weight matrix in Eq (13) is designed as a learnable parameter and initialized using Xavier uniform initialization to ensure numerical stability at the early stage of training. During training, this matrix is updated via backpropagation to adaptively capture the evolving trend across multiple time steps. Additionally, to mitigate the risk of error accumulation during multi-step trend extrapolation, we adopt a residual-style skip connection in the decoder and apply L2 regularization and early stopping. These strategies help to stabilize trend prediction and enhance robustness against long-term forecast deviations.

### 3.3 Knowledge extraction process

#### 3.3.1 Autocorrelation function.

To enhance the capability of modeling long-range dependencies in time series, we introduce the autocorrelation function (ACF) as an auxiliary mechanism. ACF measures the correlation between a time series and a lagged version of itself, allowing the model to explicitly encode repeating patterns or structural temporal regularities.

Given a time series $\{x_{t}{\}}_{t=1}^{T}$, its autocorrelation at lag *l* is defined as:

$$\text{ACF}(l)=\frac{\sum_{t=1}^{T-l}(x_{t}-\bar{x})(x_{t+l}-\bar{x})}{\sum_{t=1}^{T}(x_{t}-\bar{x}{)}^{2}}$$ (14)

where $\bar{x}$ is the mean of the time series. This function quantifies how past values influence future values at different time scales.

In our framework, ACF values are computed for each time segment and used to weight candidate connections in the attention mechanism. Specifically, when constructing the query-key similarity map, we incorporate a weighting term based on ACF to prioritize temporally correlated elements. The combined similarity score becomes:

$$S(q_{i},k_{j})=\alpha \cdot \frac{q_{i}k_{j}^{T}}{\sqrt{d}}+(1-\alpha )\cdot \text{ACF}(|i-j|)$$ (15)

where α∈[0,1] controls the balance between dot-product similarity and autocorrelation guidance.

This integration ensures that attention weights are not only based on instantaneous token-level similarity but also reflect broader temporal structure, especially beneficial for periodic or structured sequences. In summary, the ACF module enriches the temporal modeling capacity by explicitly introducing interpretable lag-based correlations into the attention computation.

#### 3.3.2 Self-attention mechanism.

The canonical self-attention mechanism is defined by the tuple inputs *Q*, *K*, and *V*, which correspond to the query, key, and value matrices, respectively. This mechanism performs scaled dot-product attention, computed as:

$$A(Q,K,V)=\text{Softmax}\left(\frac{QK^{T}}{\sqrt{d}}\right)V$$ (16)

where $Q\in {\mathbb{R}}^{L_{Q}\times d}$, $K\in {\mathbb{R}}^{L_{K}\times d}$, and $V\in {\mathbb{R}}^{L_{V}\times d}$, with *d* representing the input dimension. To further analyze the self-attention mechanism, we focus on the attention distribution of the *i*-th query, denoted as *q*_*i*_, which is based on an asymmetric kernel smoother. The attention for the *i*-th query is formulated in probabilistic terms:

$$A(q_{i},K,V)=\sum_{j}\frac{k(q_{i},k_{j})}{\sum_{j}k(q_{i},k_{j})}v_{j}={𝔼}_{p(k_{j}|q_{i})}[v_{j}]$$ (17)

where $p(k_{j}|q_{i})=\frac{k(q_{i},k_{j})}{\sum_{j}k(q_{i},k_{j})}$, and $k(q_{i},k_{j})$ represents the asymmetric exponential kernel $\exp\left(\frac{q_{i}k_{j}^{T}}{\sqrt{d}}\right).$ This self-attention mechanism combines the values and produces outputs by computing the probability $p(k_{j}|q_{i})$. However, this process involves quadratic dot-product computations, resulting in a complexity of $O(L_{Q}L_{K})$, which poses a significant limitation in memory usage, particularly for models designed to enhance predictive capacity.

#### 3.3.3 Knowledge selection.

Knowledge Selection refers to selecting the most representative queries from multiple candidates for knowledge extraction. By measuring the distance between the query distribution and a reference distribution, the relevance of each query can be evaluated, enabling the identification and utilization of important queries. From Eq (15), the attention of the *i*-th query across all keys is represented as a probability distribution $p(k_{j}|q_{i})$, where the output is computed by aggregating the values *v* weighted by this probability. High dot-product values between query-key pairs lead to a non-uniform attention distribution, as dominant query-key pairs shift the attention probability away from a uniform distribution. If $p(k_{j}|q_{i})$ closely resembles a uniform distribution, $q(k_{j}|q_{i})=\frac{1}{L_{K}}$, then the self-attention essentially produces an averaged summation over the values *v*, diminishing the significance of individual values.

To mitigate this, we introduce a knowledge extraction mechanism that evaluates the similarity between the attention probability *p* and a baseline distribution *q* using the Kullback-Leibler (KL) divergence [41,42]. This measure effectively reduces the influence of less significant queries. The similarity between *p* and *q* for the *i*-th query is computed as:

$$\text{KL}(q||p)=\ln\frac{1}{L_{K}}\sum_{j=1}^{L_{K}}e^{\frac{q_{i}k_{j}^{T}}{\sqrt{d}}}-\frac{1}{L_{K}}\sum_{j=1}^{L_{K}}\frac{q_{i}k_{j}^{T}}{\sqrt{d}}-\ln L_{K}$$ (18)

From this, we define the distillation measure *M*(*q*_*i*_,*K*) for the *i*-th query as:

$$M(q_{i},K)=\ln\left(\sum_{j=1}^{L_{K}}e^{\frac{q_{i}k_{j}^{T}}{\sqrt{d}}}\right)-\frac{1}{L_{K}}\sum_{j=1}^{L_{K}}\frac{q_{i}k_{j}^{T}}{\sqrt{d}}$$ (19)

A larger *M*(*q*_*i*_,*K*) value indicates that the *i*-th query has a more diverse attention distribution, potentially focusing on dominant dot-product pairs in the tail of the self-attention output. This approach allows the model to prioritize influential query-key pairs, thereby improving the overall effectiveness of the knowledge extraction process.

#### 3.3.4 Decoupled knowledge extraction.

**Period-based dependencies.** The period-based dependencies are quantified using the autocorrelation function, which measures the similarity between different time points in a time series, revealing its underlying periodic characteristics. For a discrete time series {*X*_*t*_}, the autocorrelation function is defined as:

$$R_{XX}(\tau )=\lim_{L\to \infty }\frac{1}{L}\sum_{t=1}^{L}X_{t}X_{t-\tau },$$ (20)

Where *τ* represents the time lag, *L* is the total length of the series, and {*X*_*t*_} and $\left\{X_{t-\tau }\right\}$ are the values at the current and lagged time points, respectively. The autocorrelation function computes the cumulative similarity over lagged time intervals, reflecting the degree of self-similarity within the series for various time delays. Peaks in the autocorrelation values indicate potential periodicity and help identify the likely period lengths.

By identifying the peaks of the autocorrelation function, the most probable period lengths $\left(\tau_{1},\tau_{2},\ldots ,\tau_{k}\right)$ can be determined. These period lengths not only capture the dominant periodic patterns in the series but also serve as weighted features, enhancing interpretability and predictive capabilities.

**Time-delay aggregation.** The time-delay aggregation method for knowledge acquisition focuses on estimating the correlation of sub-sequences within a specific period. Therefore, we propose an innovative time-delay aggregation module that can perform hierarchical convolution operations on sub-sequences based on the selected time delays $\tau_{1},\ldots ,\tau_{k}$, thereby narrowing down the key knowledge weight matrix. This process captures sub-sequences from the same location and similar positions within the period, extracting the potential key-weight aggregation matrix. Finally, we apply the Softmax function to normalize the weights, enhancing the accuracy of sub-sequence aggregation.

For a time series *x* of length *L*, after projection and filtering of the weight matrix, we obtain the query $\hat{Q}$, key *K*, and value *v*. The knowledge extraction attention mechanism is then as follows:

$$\tau_{1},\cdots ,\tau_{k}=\arg{\mathrm{Topk}}_{\tau \in \{1,\cdots ,L\}}\left({ℛ}_{𝒬,𝒦}(\tau )\right)$$ (21)

$${ℛ}_{\hat{Q},𝒦}(\tau )={\mathrm{Top}}_{u}(M(Q,K))\cdot {ℛ}_{Q,𝒦}(\tau )$$ (22)

$${ℛ}_{\hat{Q},𝒦}(\tau_{1}),\cdots ,{ℛ}_{\hat{Q},𝒦}(\tau_{k})=\mathrm{SoftMax}\left({ℛ}_{\hat{Q},𝒦}(\tau_{1}),\cdots ,{ℛ}_{\hat{Q},𝒦}(\tau_{k})\right)$$ (23)

$$\text{KEDattention}(\hat{Q},𝒦,𝒱)=\sum_{i=1}^{k}\mathrm{Roll}(𝒱,\tau_{i}){ℛ}_{\hat{Q},𝒦}(\tau_{i})$$ (24)

Where argTopk(·) is used to obtain the top *k* parameters of self-attention, and let k=⌈c×logL⌉, where *c* is a hyperparameter. ${ℛ}_{Q,K}$ represents the self-attention matrix between sequences *Q* and *K*. *Top*_*u*_ selects the most important *u* queries in the weight matrix. ${ℛ}_{\hat{Q},𝒦}$ represents the self-attention matrix after filtering between sequences *Q* and *K*. Roll(X,τ) denotes the operation of temporally shifting *X* by *τ*, where the elements shifted out from the front are reintroduced at the end. For the encoder-decoder self-attention, *K* and *V* come from the encoder $X_{\text{en}}$ and are adjusted to length *O*, with *Q* originating from the previous block of the decoder.

**Fig 3:**
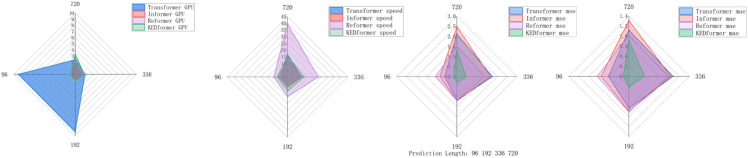
In the experiment analyzing model computational efficiency and performance, four different models are used to perform long-term time series forecasting tasks on the Exchange dataset. The input length is set to *I* = 96, and the prediction lengths are O∈{96,192,336,720}.

**Fig 4:**
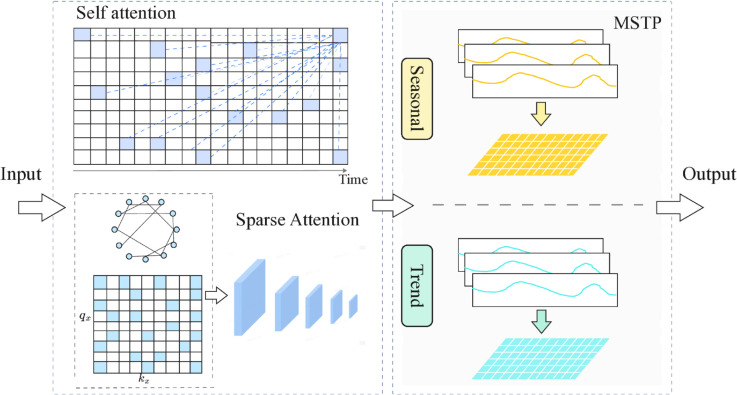
The synergistic effect of the Knowledge Extraction Attention module and the time series pooling decomposition method.

In summary, as illustrated in Fig 3, the collaborative interaction between the Knowledge Extraction Attention module and the time-series pooling decomposition method markedly enhances the predictive efficiency of KEDformer within the overall architecture (Fig 4).

## 4 Experiment

### 4.1 Datasets

In order to evaluate the effectiveness of the proposed KEDformer model, five public datasets were employed. These datasets consisted of four periodic datasets and one non-periodic dataset. They encompassed a variety of tasks and are described in detail as follows:(1) ETT [43]: This dataset comprises four sub-datasets—ETTh1, ETTh2, ETTm1, and ETTm2. The data in ETTh1 and ETTh2 were sampled every hour. Meanwhile, the data in ETTm1 and ETTm2 were sampled every 15 minutes. These datasets include load and oil temperature measurements collected from power transformers between July 2016 and July 2018.(2) Electricity [44]: This dataset contains hourly electricity consumption data from 321 customers. The data span from 2012 to 2014.(3) Exchange Rates [45]: This non-periodic dataset records the daily exchange rates of eight countries. It spans the years 1990 to 2016.(4) Traffic [46]: This dataset consists of hourly traffic data from the California Department of Transportation. It captures road occupancy through various sensors on the Bay Area Highway.(5) Weather [47]: This dataset includes meteorological data recorded every 10 minutes throughout the year 2020. There are 21 indicators such as temperature and humidity.In accordance with standard protocols, all datasets were chronologically split into training, validation, and test sets. The ETT dataset was partitioned using a 6:2:2 ratio [43]. Meanwhile, the other datasets followed a 7:1:2 split [44–47].

### 4.2 Implementation details

Under the experimental configuration summarized in Table 2, this study embeds residual connections into the decomposition module of the Transformer-based model [32], thereby enhancing its capability to model time series, smoothing periodic fluctuations, and emphasizing long-term trends. The model is optimized using the L2 loss function and the ADAM optimizer [48], with a fixed random seed set to ensure reproducibility. Hyperparameters are systematically tuned on the validation set via a grid search strategy [49]. Specifically, we evaluated all combinations of key parameters and adopted MSE and MAE as performance metrics, with the final optimal configuration summarized in Table 3.

**Table 2:** Description of the experimental environment.

Component	Description
System	PyTorch2.7.0-Ubuntu 22.04
CPU	10vcpu
Graphics Memory	60GB
GPU	NVIDIA GeForce RTX 3090 (24 GB)
CUDA	12.4
Python	3.10.14
Pytorch	2.7.0

**Table 3:** Table of optimal hyperparameter settings.

Parameter	Value
seq_len	96
label_len	48
d_model	512
dropout	0.1
batch_size	32
encoder layer	2
decoder layer	1
learning rate	0.0001

To mitigate overfitting and improve generalization, two complementary strategies were employed: (1) an early stopping mechanism, in which training was terminated if validation performance did not improve for 10 consecutive epochs; and (2) regularization, achieved by introducing dropout in critical layers and applying a weight decay term of 0.1. These measures effectively reduced the risk of over-parameterization and enhanced the robustness of the model in long-horizon forecasting tasks.

It is noteworthy that long-term sequence forecasting is inherently more prone to overfitting than short-term tasks, as the model must simultaneously capture local perturbations and global trends over extended horizons. Excessive reliance on training-specific patterns may therefore impair generalization. To address this issue, we further assessed model robustness through repeated experiments with different random seeds. Specifically, all experiments were conducted three times, and the mean and standard deviation were reported to ensure the reliability and reproducibility of the results.

### 4.3 Baselines

We evaluated eleven representative baseline models for time-series forecasting. Among them, Transformer-based models include Autoformer [32], Informer [17], Reformer [50], Pyraformer [51], FEDformer [28], and LogTrans [18]; recurrent neural network (RNN)–based models comprise LSTNet [52], LSTM [53], and DeepAR [54]; the convolutional neural network (CNN)–based model is TCN [55]; and statistical decomposition and linear models include Prophet [56] and ARIMA [57]. These bas [57]elines cover a broad spectrum of mainstream forecasting paradigms—from long-range dependency modeling and frequency-domain decomposition to seasonal-trend analysis—thereby providing a comprehensive and systematic benchmark for this study.

### 4.4 Performance comparison

The Mean Squared Error (MSE) emphasizes large deviations by penalizing them more heavily, while the Mean Absolute Error (MAE) provides a more intuitive measure of the average prediction bias. As both metrics are widely used and complementary in time series forecasting tasks, we adopt MSE and MAE as the two primary indicators to evaluate the predictive accuracy of our model. Their formulations are given in (25 and 26):

$$\text{MSE}=\frac{1}{n}\sum_{i=1}^{n}{\left(y_{x}-{\hat{y}}_{i}\right)}^{2}$$ (25)

$$\text{MAE}=\frac{1}{n}\sum_{i=1}^{n}\left|y_{x}-{\hat{y}}_{i}\right|$$ (26)

In the formulas of MSE and MAE *n* is the total number of samples. *y*_*x*_ is the true value of the *x*-th sample. ${\hat{y}}_{x}$ is the predicted value of the *x*-th sample.

In multi-step forecasting tasks, errors are first computed at each prediction horizon and then averaged across the entire forecast range. Let *O* denote the prediction length, *N* the number of test samples, and *D* the number of variables. The aggregated Mean Squared Error (MSE) and Mean Absolute Error (MAE) are formulated as follows:

$$\text{MSE}=\frac{1}{N\cdot O\cdot D}\sum_{i=1}^{N}\sum_{t=1}^{O}\sum_{d=1}^{D}{\left(y_{i,t,d}-{\hat{y}}_{i,t,d}\right)}^{2}$$ (27)

$$\text{MAE}=\frac{1}{N\cdot O\cdot D}\sum_{i=1}^{N}\sum_{t=1}^{O}\sum_{d=1}^{D}\left|y_{i,t,d}-{\hat{y}}_{i,t,d}\right|$$ (28)

Here, *y*_*i*,*t*,*d*_ and ${\hat{y}}_{i,t,d}$ represent the ground-truth and predicted values of the *i*-th sample at forecasting step *t* and variable dimension *d*, respectively. For each dataset, the final MSE and MAE are obtained by aggregating across all time steps and all variables in the test set.

#### 4.4.1 Multivariate results.

In multivariate long-horizon forecasting, KEDformer demonstrates consistent stability and superior accuracy across all benchmark datasets Table 4. Under the input-96-predict-336 setting, KEDformer achieves substantial gains on five real-world datasets: it attains the best MSE in 11 out of 20 comparisons and the best MAE in 8 out of 20, indicating robust performance across data regimes and horizons.

**Table 4:** Multivariate results.

Model		KEDformer		Autoformer [16]		Informer [17]		Reformer [50]		LSTNet [52]		LSTM [53]		TCN [55]		Transformer [22]		Pyraformer [51]		FEDformer [28]	
Metric		MSE	MAE	MSE	MAE	MSE	MAE	MSE	MAE	MSE	MAE	MSE	MAE	MSE	MAE	MSE	MAE	MSE	MAE	MSE	MAE
**Exchange [28]**	**96**	**0.142**	**0.273**	0.197	0.323	0.847	0.752	1.065	0.829	1.551	1.058	1.453	1.049	3.004	1.432	0.835	0.710	0.640	0.631	0.151	0.281
	**192**	**0.271**	0.380	0.300	**0.369**	1.204	0.895	1.188	0.906	1.477	1.028	1.846	1.179	3.048	1.444	1.254	0.871	0.823	0.731	0.321	0.453
	**336**	**0.456**	**0.506**	0.509	0.524	1.672	1.036	1.357	0.976	1.507	1.031	2.136	1.231	3.113	1.459	1.644	1.035	1.465	0.973	0.508	0.626
	**720**	**1.089**	**0.811**	1.447	0.941	2.478	1.310	1.510	1.016	2.285	1.243	2.984	1.427	3.150	1.458	2.375	1.292	1.602	1.019	1.236	0.863
**Traffic [28]**	**96**	**0.409**	0.379	0.613	0.388	0.719	0.391	0.732	0.423	1.107	0.685	0.843	0.453	1.438	0.784	0.664	0.364	0.652	0.362	0.573	**0.353**
	**192**	0.607	0.380	0.616	0.382	0.696	0.379	0.733	0.420	1.157	0.706	0.847	0.453	1.463	0.794	0.663	0.365	0.655	**0.359**	**0.599**	0.375
	**336**	0.619	**0.323**	0.622	0.337	0.777	0.420	0.742	0.420	1.216	0.730	0.853	0.455	1.479	0.799	0.674	0.360	0.695	0.392	**0.608**	0.380
	**720**	0.656	0.403	0.660	0.408	0.864	0.472	0.755	0.423	1.481	0.805	1.500	0.804	1.499	0.804	0.661	**0.368**	0.687	0.405	**0.610**	0.378
**ETTm2 [28]**	**96**	**0.234**	**0.316**	0.255	0.339	0.365	0.453	0.658	0.619	3.142	1.365	2.041	1.073	3.041	1.330	0.463	0.505	0.385	0.458	0.235	0.318
	**192**	**0.278**	0.338	0.281	0.340	0.533	0.563	1.078	0.827	3.154	1.369	2.249	1.112	3.072	1.339	1.173	0.786	0.811	0.696	1.124	**0.244**
	**336**	0.336	0.369	0.339	0.372	1.363	0.887	1.549	0.972	3.160	1.369	2.568	1.238	3.105	1.348	1.319	0.879	1.315	0.852	**0.188**	**0.296**
	**720**	0.417	0.414	0.422	0.419	3.379	1.388	2.631	1.242	3.171	1.368	2.720	1.287	3.135	1.354	3.313	1.355	4.584	1.630	**0.245**	**0.340**
**Weather [28]**	**96**	0.265	0.333	0.266	0.336	0.332	0.368	0.689	0.596	0.594	0.587	0.560	0.565	0.615	0.589	0.408	0.422	**0.178**	**0.257**	0.222	0.303
	**192**	0.305	0.364	0.307	0.367	0.598	0.544	0.752	0.638	0.597	0.587	0.639	0.608	0.629	0.600	0.582	0.550	**0.230**	**0.314**	0.284	0.352
	**336**	0.359	0.399	0.359	0.395	0.702	0.620	0.639	0.596	0.597	0.587	0.455	0.454	0.639	0.608	0.623	0.563	**0.289**	**0.359**	0.420	0.553
	**720**	0.414	0.423	0.419	0.428	0.831	0.731	1.130	0.792	0.618	0.599	0.535	0.520	0.618	0.599	0.892	0.704	0.410	0.434	**0.406**	**0.419**
**Electricity [28]**	**96**	**0.201**	0.317	0.201	0.317	0.274	0.368	0.312	0.402	0.680	0.645	0.985	0.813	0.615	0.784	0.255	0.352	0.303	0.377	0.201	**0.315**
	**192**	**0.219**	**0.330**	0.222	0.334	0.296	0.368	0.348	0.433	0.725	0.676	0.995	0.824	0.985	0.824	0.268	0.368	0.298	0.385	0.221	0.333
	**336**	**0.229**	**0.336**	0.231	0.338	0.300	0.394	0.350	0.433	0.828	0.727	1.000	0.824	1.000	0.824	0.272	0.367	0.346	0.416	0.236	0.425
	**720**	**0.253**	**0.361**	0.253	0.361	0.373	0.439	0.340	0.420	0.957	0.811	1.438	0.784	1.438	0.784	0.288	0.374	0.313	0.400	0.268	0.380
**Count**		11	8	0	1	0	0	0	0	0	0	0	0	0	0	0	1	3	4	6	6

Multivariate results with different prediction lengths O∈{96,192,336,720} for five different datasets when *I* = 96. To summarize the results, we counted the number of times each model achieved the best performance. The best average results are in **bold**, while the second-best results are underlined.

In contrast, the baselines exhibit structural limitations under long horizons, multivariate coupling, and pronounced non-stationarity. Transformer incurs quadratic complexity with sequence length and lacks mechanisms tailored to temporal non-stationarity (i.e., evolving data statistics due to seasonality, policy shifts, or holidays), leading to slow adaptation and accumulated error. Informer, Reformer, Pyraformer, and FEDformer gain efficiency via attention sparsification or downsampling, which narrows effective context and increases the risk of missing weak yet critical long-range dependencies; frequency-domain pipelines further introduce phase and amplitude distortions when reconstructing to the time domain. LSTNet, LSTM, and TCN are constrained in modeling very long dependencies and cross-variable interactions: RNNs suffer gradient decay, TCNs remain locally biased and less robust to phase shifts, and LSTNet’s linear residual path under-represents nonlinear cross-variable relations, leading to underfitting.

KEDformer’s advantage stems from the integration of an explicit autocorrelation module and fidelity-oriented near-linear sparse attention. Autocorrelation explicitly retrieves repeated patterns and latent dependencies across variables and temporal lags, while sparse attention suppresses redundancy and preserves salient channels and dominant delays. Additionally, the MSTP decomposition jointly models local perturbations and global trends, strengthening representation of complex temporal structure. Together, these components—trained end-to-end—yield superior accuracy and robustness in multivariate forecasting.

#### 4.4.2 Univariate results.

In the univariate forecasting setting, we selected the representative ETTm2 and Exchange datasets for evaluation, as they respectively exhibit strong periodic industrial characteristics and highly volatile financial behaviors. This complementary design enables a comprehensive assessment of the model’s robustness and generalization across diverse time series scenarios, with the results summarized in Table 5.

**Table 5:** Univariate results.

Model		KEDformer		Autoformer [16]		LogTrans [52]		Informer [17]		Reformer [50]		DeepAR [57]		Prophet [34]		ARIMA [36]		Transformer [22]		Pyraformer [51]		FEDformer [28]	
Metric		MSE	MAE	MSE	MAE	MSE	MAE	MSE	MAE	MSE	MAE	MSE	MAE	MSE	MAE	MSE	MAE	MSE	MAE	MSE	MAE	MSE	MAE
**ETTm2 [28]**	**96**	**0.064**	**0.187**	0.065	0.189	0.088	0.225	0.082	0.217	0.131	0.288	0.099	0.253	0.287	0.456	0.211	0.362	0.120	0.243	0.117	0.246	0.118	0.248
	**192**	**0.114**	**0.251**	0.118	0.256	0.132	0.283	0.133	0.284	0.186	0.354	0.154	0.304	0.312	0.483	0.261	0.406	0.179	0.313	0.185	0.317	0.198	0.333
	**336**	**0.147**	**0.297**	0.147	0.305	0.180	0.336	0.201	0.361	0.220	0.381	0.277	0.428	0.428	0.593	0.317	0.448	0.237	0.369	0.284	0.401	0.298	0.408
	**720**	**0.181**	**0.333**	0.182	0.335	0.300	0.435	0.269	0.407	0.267	0.430	0.332	0.468	0.534	0.593	0.366	0.487	0.316	0.429	0.363	0.466	0.392	0.484
**Exchange [28]**	**96**	0.161	0.309	0.241	0.387	0.591	0.615	0.279	0.441	1.327	0.944	0.417	0.515	0.828	0.762	**0.112**	**0.245**	0.318	0.419	0.331	0.409	0.283	0.395
	**192**	**0.203**	**0.356**	0.273	0.403	1.183	0.912	0.315	0.498	1.258	0.924	0.813	0.735	0.909	0.974	0.304	0.404	0.359	0.438	0.332	0.408	0.290	0.390
	**336**	0.489	0.497	0.508	0.539	1.367	0.984	2.438	1.048	1.262	1.296	1.331	0.962	1.304	0.988	0.736	0.598	0.479	0.510	0.443	0.465	**0.355**	**0.441**
	**720**	0.896	0.724	0.991	0.768	1.872	1.072	2.010	1.181	1.280	0.953	1.894	1.181	3.238	1.566	1.871	0.935	**0.445**	**0.498**	0.505	0.511	0.476	0.519
**Count**		5	5	0	0	0	0	0	0	0	0	0	0	0	0	1	1	1	1	0	0	1	1

Univariate results with different prediction lengths O∈{96,192,336,720} for two different datasets when *I* = 96. The best average results are in **bold**, while the second-best results are in underlined.

Compared with multiple baselines, KEDformer achieves overall state-of-the-art performance in long-term forecasting. Under the input-96-predict-336 configuration, it delivers the best results in 5 out of 8 cases for both Mean Squared Error (MSE) and Mean Absolute Error (MAE). To further enrich the baseline pool, LogTrans, DeepAR, Prophet, and ARIMA were additionally included in the univariate experiments. The results highlight their inherent limitations: LogTrans, while employing logarithmic sparse attention to reduce computational cost, fails to capture long-term dependencies and non-periodic patterns, leading to error accumulation over extended horizons; DeepAR, as an autoregressive RNN-based method, relies on step-by-step predictions, hindering parallelism and amplifying errors in long sequences; Prophet and ARIMA, constrained by linear decomposition and stationarity assumptions, lack adaptability to nonlinear dynamics and multi-scale temporal variations.

By contrast, KEDformer excels through its autocorrelation mechanism for enhanced global dependency modeling, sparse attention for effective redundancy filtering, and trend–seasonal decomposition module for precise temporal structure learning, thereby fully leveraging its representational advantages in low-dimensional sequences.

#### 4.4.3 Ablation research.

To systematically evaluate the impact of the proposed Knowledge Extraction and Decomposition Attention (KEDA) module on model performance, we conducted a set of ablation experiments. Three model variants were designed for comparison:

- KEDformer:which completely replaces the original self-attention and cross-attention mechanisms with KEDA;
- KEDformer V1:which replaces only the self-attention mechanism with KEDA while retaining the original cross-attention;
- KEDformer V2: which uses conventional attention mechanisms in both positions.

These models were evaluated on the *Exchange* and *Weather* datasets, and the results are shown in Table 6. KEDformer outperformed its counterparts in 14 out of 16 test cases, while KEDformer V1 showed improvements in only 2 cases. The results demonstrate the significant advantage of KEDA as a unified attention mechanism.The KEDA module integrates an autocorrelation mechanism with a sparse attention strategy: the autocorrelation component precisely captures key dependencies across time segments, enhancing the model’s temporal modeling capability; meanwhile, the sparse attention mechanism sparsifies the attention weight matrix, effectively reducing interference.

**Table 6:** Ablation results.

Model		KEDformer		KEDformerV1		KEDformerV2		Informer [17]		Reformer [50]	
**Self-att**		KEDatt		KEDatt		KEDatt-f		ProbAtt		Reatt	
**Cross-att**		KEDatt		KEDatt-f		KEDatt-f		ProbAtt		Reatt	
**Metric**		**mse**	**mae**	**mse**	**mae**	**mse**	**mae**	**mse**	**mae**	**mse**	**mae**
**Exchange [45]**	**96**	**0.142**	**0.273**	0.175	0.318	0.170	0.315	0.847	0.752	1.065	0.829
	**192**	**0.271**	**0.380**	0.281	0.387	0.302	0.407	1.204	0.895	1.188	0.906
	**336**	**0.456**	**0.506**	0.472	0.514	0.502	0.535	1.672	1.036	1.357	0.976
	**720**	1.089	0.811	**1.094**	**0.798**	1.097	0.821	2.478	1.310	1.510	1.016
**Weather [47]**	**96**	**0.265**	**0.333**	0.277	0.360	0.354	0.382	0.384	0.458	0.689	0.596
	**192**	**0.305**	**0.364**	0.418	0.467	0.359	0.417	0.544	0.652	0.752	0.638
	**336**	**0.359**	**0.399**	0.482	0.505	0.518	0.523	0.794	0.794	0.639	0.596
	**720**	**0.414**	**0.423**	0.554	0.544	0.645	0.604	0.741	0.869	1.130	0.792
**Count**		7	7	1	1	0	0	0	0	0	0

The ablation study was conducted by systematically modulating the attention mechanisms of KEDformer, thereby generating multiple model variants. The best average results are in **bold**, while the second-best results are underlined.

## 5 Discussion

### 5.1 Time series decomposition effects on the mode

**Fig 5:**
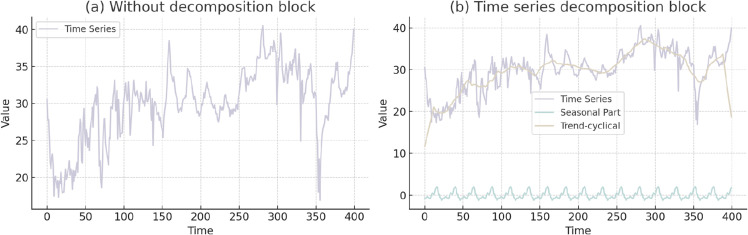
Visualization of time series decomposition. In the left subfigure (a), the raw time series data is shown without decomposition, displaying interwoven fluctuations and trends. In contrast, the right subfigure (b) presents the time series decomposed into three components: the original time series in purple, the trend-cyclical component in beige, and the seasonal component in teal.

The time series decomposition applied to the ETTm1 dataset has clearly revealed distinct seasonal and trend-cyclical patterns, enabling the KEDformer model to more accurately capture periodic variations and long-term trends within the data. By effectively separating seasonal patterns from trend components, the integration of decomposition has significantly enhanced the model’s predictive accuracy. Focusing on short-term fluctuations while retaining an understanding of long-term trends, the model is better equipped for accurate forecasting. As illustrated in Fig 5, these improvements can be attributed to several key factors: First, the explicit modeling of seasonal variations allows the model to more effectively adapt to recurring patterns, thereby strengthening its ability to project future values based on historical data. Second, the decomposition process helps identify significant features within the data, enabling the model to prioritize relevant information during the forecasting process.

### 5.2 Effect of KEDformer number on encode and decode

In this study, we conducted comparative experiments using the Exchange dataset. We varied the number of KEDformer mechanisms in these experiments. The results, illustrated in Fig 5, demonstrate that the model achieves superior performance when the number of KEDformer mechanisms in the decoding phase exceeds that in the encoding phase. This improvement can be attributed to the model’s enhanced ability to focus on the most informative features during the decoding process. As a result, it effectively captures dependencies between the predicted outputs and historical inputs. Conversely, performance declines when the number of KEDformer mechanisms in the decoding phase equals that in the encoding phase.

### 5.3 Effect of KEDformer on computational efficiency

**Fig 6:**
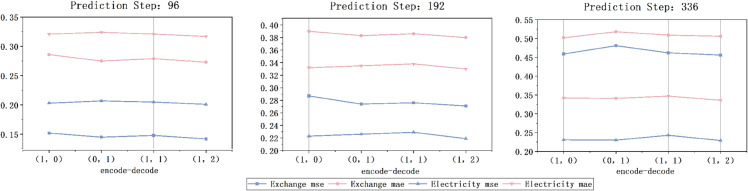
Visualization of time series decomposition results. In a comparative experiment that controls the number of KEDformer mechanisms during the encoding and decoding processes, we set the input length *I* = 96 and the prediction lengths O∈{96,192,336}.

We conducted experiments to evaluate the impact of increasing the number of KEDformer mechanisms on the computational efficiency of the model, as shown in Fig 6. The results demonstrate that the model achieves improved efficiency as the number of KEDformer mechanisms increases across various datasets, including ETTm1, ETTm2, and Weather. Notably, the time required for each epoch decreases significantly with the increase in the number of KEDformer mechanisms. The most pronounced improvement is observed in the ETTm1 dataset, where computation time drops from 794.0 seconds to 467.2 seconds. This enhancement can be attributed to the model’s improved ability to capture temporal dependencies and optimize resource utilization. This enables parallel processing and more effective distribution of the computational load.

### 5.4 Efficiency analysis and performance analysis

**Fig 7:**
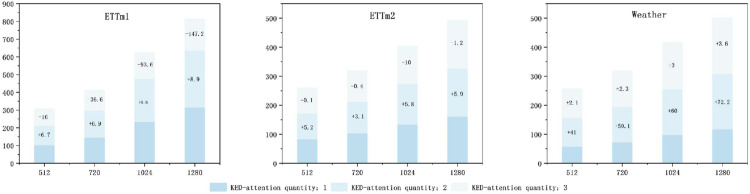
Impact of KEDattention mechanisms on model computational efficiency. The input length is set to *I* = 96, and the prediction steps are O={512,720,1024,1280}. The time required for each epoch is used as an indicator of the model’s computational speed.

In this study, we conducted a comprehensive analysis of the computational efficiency and predictive performance of models employing different self-attention mechanisms, with the results presented in Fig 7. On the Exchange dataset,The KEDformer model ranks second in terms of memory usage (measured in GB), which is primarily attributed to its sparse attention mechanism. The sparse attention mechanism is one of the key technologies for reducing computational complexity and memory consumption. By selecting only the key positions in the input sequence for attention calculation instead of performing global calculations for all positions, this mechanism reduces the computational complexity from the traditional *O*(*L*^2^) to O(LlogL), significantly decreasing memory usage. the KEDformer model ranked third in terms of running time but achieved the highest prediction accuracy. This superior performance can be primarily attributed to its optimized knowledge extraction mechanism and seasonal trend decomposition approach, which significantly enhance the model’s ability to capture key patterns in time series data. However, when dealing with time series data lacking clear periodicity, the model’s performance may deteriorate, as the seasonal trend decomposition may fail to effectively extract relevant information. Additionally, an improper configuration of the number of KEDformer mechanisms can reduce computational efficiency and negatively impact the final prediction results. In this study, computational efficiency was assessed by measuring the time required for each model to complete a training epoch (in seconds), while predictive performance was evaluated using the Mean Squared Error (MSE) and Mean Absolute Error (MAE).

### 5.5 Computation efficiency

In the multivariate setting and with the current optimal implementation of all methods, KEDformer has achieved a significant enhancement in computational efficiency compared to conventional Transformer models. This improvement effectively addresses the challenges associated with the quadratic time complexity O(L2) and memory usage O(L2) inherent to standard self-attention mechanisms. By employing sparse attention and autocorrelation strategies, KEDformer reduces both the time complexity and memory usage to O(LlogL), thereby enhancing the model’s capability to process long sequence data. As illustrated in Fig 7, KEDformer maintains its time and memory complexity while significantly improving prediction accuracy, enabling the model to handle longer sequences more efficiently. During the testing phase, KEDformer completes predictions in a single step, in contrast to traditional models that require O(L) steps, thereby substantially increasing its efficiency. As demonstrated in Table 7, KEDformer strikes a superior balance between computational efficiency and predictive accuracy, rendering it a practical solution for long-term time series forecasting tasks in resource-constrained environments.

**Table 7:** Complexity analysis of space and time for different forecasting models.

Methods		KEDformer	Autoformer [16]	Informer [17]	LogTrans [18]	Transformer [58]	LSTM [52]
Training	Time	𝒪(LlogL)	𝒪(LlogL)	𝒪(LlogL)	𝒪(LlogL)	$𝒪(L^{2})$	𝒪(L)
	Memory	𝒪(LlogL)	𝒪(LlogL)	𝒪(LlogL)	$𝒪(L^{2})$	$𝒪(L^{2})$	𝒪(L)
Testing	Steps	1	1	1	1	*L*	*L*

## 6 Conclusion

This study proposes KEDformer, a novel and efficient framework for long-term time series forecasting tasks. By introducing a sparse attention mechanism, the model reduces the quadratic complexity of standard self-attention to near-linear complexity, thereby significantly improving processing speed for long sequences. In addition, KEDformer integrates seasonal–trend decomposition with an autocorrelation mechanism to jointly model short-term perturbations and long-term structures, effectively mitigating information loss and producing forecasts that better align with real-world temporal dynamics.

Extensive experiments on multiple public benchmark datasets demonstrate that KEDformer consistently outperforms mainstream Transformer-based models in terms of long-term prediction accuracy, stability, and generalization ability, highlighting its strong adaptability across diverse forecasting tasks. Notably, for typical periodic data, the decomposition and autocorrelation modules provide substantial modeling advantages; however, in certain non-periodic or highly volatile scenarios (e.g., financial exchange rates), the effectiveness of these mechanisms is relatively constrained. This limitation is not inherent to the model itself but reflects the varying sensitivities of different data structures to specific pattern extraction methods.

To further enhance the model’s generality and flexibility, future work will explore dynamic structure selection mechanisms based on data characteristics, enabling adaptive adjustment of modeling strategies. In addition, we plan to investigate more interpretable sparse attention strategies to improve the identification of critical features and redundancy filtering in non-periodic scenarios. These directions are expected to broaden the applicability of KEDformer to a wider range of time series modeling tasks.

Overall, KEDformer marks an important step forward in long-term time series forecasting, offering a promising pathway to address the trade-off between efficiency and accuracy under high-dimensional input conditions. For example, in healthcare monitoring scenarios, KEDformer can capture both long-term trends and short-term fluctuations in physiological signals, thereby assisting in disease risk prediction. This illustrates that its applicability extends beyond standard benchmark tasks and can be adapted to more complex real-world domains. With continued structural optimization and mechanism refinement, its scope is expected to expand to increasingly diverse and challenging forecasting applications.
